# Imaging and Laboratory Results as Predictors of the Course of COVID-19

**DOI:** 10.3390/arm93040022

**Published:** 2025-07-01

**Authors:** Ewelina Tobiczyk, Hanna Maria Winiarska, Daria Springer, Aleksandra Ludziejewska, Ewa Wysocka, Szymon Skoczyński, Szczepan Cofta

**Affiliations:** 1 Department of Pulmonology, Allergology and Pulmonary Oncology, Poznan University of Medical Sciences, 60-569 Poznan, Poland; 2 Department of Laboratory Diagnostics, Poznan University of Medical Sciences, 60-569 Poznan, Poland; 3 Department of Lung Diseases and Tuberculosis, Faculty of Medical Sciences in Zabrze, Medical University of Silesia, 40-752 Katowice, Poland

**Keywords:** bilevel positive airway pressure, chest high-resolution computed tomography, continuous positive airway pressure, high-flow nasal oxygen therapy, laboratory tests, NIRS, non-invasive respiratory support, SARS-CoV-2, post-COVID-19 mortality

## Abstract

**Highlights:**

**What are the main findings?**

- Inflammatory changes involving more than 50% of the lung parenchyma on chest CT scans proved to be the best predictor of severe COVID-19 disease.
- The results of imaging and laboratory tests are useful in predicting the need for non-invasive ventilation support.

**What is the implication of the main finding?**

- Chest CT and laboratory tests should be performed upon hospital admission in patients with COVID-19
- Despite the decreased incidence of COVID-19 following the pandemic, early identification of patients at risk for severe infection remains essential.

**Abstract:**

Background: COVID-19 most often affects the respiratory system and may manifest as acute respiratory failure requiring the use of non-invasive respiratory support (NIRS). The aim of this study was to find predictors based on laboratory results and chest computed tomography (CT) scans performed on admission to the hospital indicating the need for NIRS and predicting mortality after hospital discharge. Methods: We retrospectively analysed data from consecutive patients hospitalised in the Pulmonology Department of the Temporary COVID Hospital in Poznan from 1 February 2021 to 31 March 2022. Upon admission to the department, the patients underwent a series of laboratory blood tests and high-resolution chest CT scan. Results: The study group included 282 patients, with an average age of 60.0 ± 15.0 years. In total, 54 (53%) patients of 101 requiring NIRS died from various causes or required intubation. Patients who required NIRS were significantly older and had more severe changes in the lung parenchyma. They had higher white blood cell and neutrophil counts and lower lymphocyte counts, as well as higher concentrations of D-dimer, CRP, PCT, and IL-6 and greater activities of LDH and AST. Conclusions: Laboratory tests and chest CT performed on hospital admission may be useful to rapidly identify patients at higher risk for severe disease.

## 1. Introduction

Infection caused by coronavirus, which was detected in Wuhan, China in December 2019, quickly spread around the world, and in March 2020 the World Health Organization declared a pandemic [1]. The virus was named severe acute respiratory syndrome coronavirus 2 (SARS-CoV-2), and the infection it caused was named coronavirus disease 2019 (COVID-19).

Many organs and systems are affected by SARS-CoV-2 infection, but the most common pathology requires hospitalisation as a result of respiratory infection [2]. So far, it has been found that different factors influence the course of the disease, including age, comorbidities, immune response, radiological findings, laboratory markers, and indicators of organ dysfunction [3]. New predictors of COVID-19 severity are still being sought. Research is focused on the variability of the host metabolome following SARS-CoV-2 infection [4,5]. A promising direction of research is also the search for biomarkers of neutrophil extracellular traps (NETs) and markers based on microRNA [6,7,8].

The consequence of inflammatory changes in the lung parenchyma, is the occurrence of acute respiratory failure. Basic treatment conducts passive oxygen therapy, including high-flow nasal oxygen therapy (HFNOT) and respiratory support with continuous positive airway pressure (CPAP) or bilevel positive airway pressure (BPAP) [9,10]. All these methods of oxygen therapy and ventilation support are called non-invasive respiratory support (NIRS). The use of NIRS was necessary in patients with severe COVID-19. Before the COVID-19 pandemic, NIRS was already widely used in the treatment of acute hypoxemic respiratory failure [11]. Previous studies have shown that the use of various NIRS methods in patients with COVID-19 is safe, reduces the need for intubation and may be associated with better treatment outcomes; however, when NIRS delays intubation, it may worsen patient prognosis [12,13,14,15]. In addition, NIRS may improve outcomes in patients with severe respiratory failure who are not considered suitable for escalation to invasive ventilation [16].

The long-term consequences of COVID-19 remain largely unclear. In the paediatric population, the presence of comorbidities and obesity have been found to be significant predictors of mortality in children with COVID-19 within six months of hospital discharge [17]. In the adult population, severe SARS-CoV-2 pneumonia has not been found to be associated with increased mortality after hospital discharge [18].

The aim of our study was to find predictors based on laboratory results and chest computed tomography scans performed on admission of adult patients to the hospital for the need for NIRS. We also looked for factors predicting failure of NIRS—patient death from any cause or the need for invasive mechanical ventilation (IMV). Furthermore, in patients who survived hospitalisation due to SARS-CoV-2 infection, we examined factors influencing their post-hospital mortality.

## 2. Materials and Methods

### 2.1. Study Design

We conducted a retrospective analysis of data from consecutive patients who were hospitalised in the Pulmonology Department of the Temporary COVID Hospital in Poznan between 1 February 2021 and 31 March 2022. The study was approved by the Ethics Committee of the Poznan University of Medical Science (number KB—987/2023, date 20 December 2023). Patients admitted to the department had SARS-CoV-2 infection confirmed by rapid antigen test or real time polymerase chain reaction test (PCR) and were subjected to isolation in accordance with the current Regulation of the Polish Council of Ministers. Upon admission to the ward, patients had laboratory blood tests and chest computed tomography (CT) performed. The exclusion criteria were missing data on the oxygen therapy methods used, transfer to the pulmonology department from another hospital or department, and lack of full laboratory test results and lung CT scans. Finally, only patients with complete data were included in the study.

### 2.2. Laboratory Tests

Laboratory tests were performed at time of admission: routine haematology tests, creatinine, D-dimers, aspartate aminotransferase (AST), alanine aminotransferase (ALT), lactate dehydrogenase (LDH), C-reactive protein (CRP), procalcitonin (PCT), and interleukin-6 (IL-6).

A complete blood count (CBC) was analysed using the ADVIA 2120i automated haematology analyser (Siemens Healthineers—Erlangen, Germany). D-dimer concentrations were measured with the SYSMEX CS-2500 automated analyser (Sysmex Europe SE—Norderstedt, Germany). The concentrations and activities of specific biochemical blood parameters were assessed using the Atellica automated biochemistry and immunochemistry analyser (Siemens Healthineers—Erlangen, Germany).

All tests were conducted in accordance with Good Laboratory Practice standards at the Clinical Laboratory of the University Clinical Hospital in Poznan.

### 2.3. Computed Tomography

All COVID-19 patients underwent high-resolution computed tomography (HRCT) of the chest within 48 h of hospital admission. The procedure was performed without intravenous contrast in the supine position using a 64-channel multidetector CT scanner (Revolution Frontier, 2020). The percentage of lung parenchyma involvement by the disease process was assessed by a radiologist for each patient.

Typical chest CT features in COVID-19 include ground-glass or reticular opacities with or without consolidation. Bilateral lung involvement is most common, usually with peripheral, subpleural, and posterior distribution. CT images were reviewed by experienced radiologists.

### 2.4. Patients

The study group included 282 patients aged between 25 and 92 years, with a mean age of 60.0 ± 15.0 years. Males accounted for 72% (192) of the patients enrolled into the study. Most patients admitted to the Pulmonology Department presented a severe clinical condition or comorbidities related to the respiratory system. However, admission depended on the availability of vacancies in the Temporary Hospital Departments. Overall, 101 patients required NIRS, and in 181 patients passive oxygen therapy was sufficient. In total, 54 patients of 101 requiring NIRS died from various causes or required intubation and treatment in the Intensive Care Unit (ICU). After hospitalisation due to SARS-CoV-2 infection, 229 patients were discharged, including 158 men (69%) and 71 women (31%). The mean hospitalisation time was 10 days. There was no statistically significant difference between the duration of hospitalisation and the mean age of women and men.

### 2.5. Oxygen Supplementation

The method of oxygen therapy was selected depending on the clinical state and oxygen saturation level. The methods of passive oxygen therapy used include nasal cannula, simple mask, reservoir mask, HFNOT, and active oxygen therapy (CPAP and BPAP). Individual methods of oxygen therapy were applied in accordance with the guidelines presented by Czajkowska-Malinowska et al. [10].

Passive oxygen therapy was gradually escalated depending on the patient’s oxygen requirements. It was started successively with oxygen cannulas, a simple mask, a mask with a reservoir, and HFNOT. However, if the use of these passive oxygen therapy methods did not allow maintaining saturation in the range of ≥90–92%, and/or the patient’s respiratory effort was visible, active oxygen therapy methods were used. Active oxygen therapy methods in Pulmonology Department were used non-invasively. If, despite the use of NIRS, patients experienced worsening hypoxemia, dyspnoea, tachypnoea, or impaired consciousness, intubation and IMV were necessary. Invasive ventilation was performed in the ICU. Qualification for treatment in the ICU was based on the Guidelines of the Polish Society of Anaesthesiology and Intensive Care specifying the qualification rules and criteria for admitting patients to the Departments of Anaesthesiology and Intensive Care [19]. When patients were in a serious clinical state and the doctors determined that due, to their age, comorbidities, and general health condition, they would not benefit from intubation and invasive ventilation, further treatment was conducted at the Pulmonology Department.

### 2.6. Statistical Analysis

Descriptive statistics were applied to summarise the key demographic characteristics, as well as the laboratory and CT results, of all patients in the study. To assess the normality of the analysed variables, we used the Shapiro–Wilk test. For variables that had a normal distribution, we presented the result as the mean with standard deviation (SD), and variables that did not have a normal distribution were presented as the median with interquartile ranges (Q1–Q3). Since the data did not follow a normal distribution, continuous variables were compared using the Mann–Whitney U test. Categoric variables were presented as numbers and percentages and were compared by chi-square test between the study groups. Univariate logistic regression analyses was conducted to identify the factors associated with the need to use NIRS. Variables whose *p* values in univariate analysis were less than 0.05 were entered into multivariate logistic regression analysis to identify a risk factor model. Survival of patients after hospital discharge was analysed using the Kaplan–Meier method. The log-rank test was used to compare the two groups for each predictor. We calculated time to death as the time in months between hospital discharge and date of death, and for survivors we ended follow-up on 18 April 2025. Crude and adjusted hazard ratios (HRs) with 95% confidence intervals (CIs) were calculated from Cox regression for variables that were found to be significant in the survival analyses using Kaplan–Meier curves. The adjusted model, which included age and the need for NIRS, showed the best predictive value for estimating survival after hospitalisation due to COVID-19. The selection of the best model was based on the value of the R2 coefficient and information criteria.

The level of significance was set at *p* < 0.05. Statistical analysis was carried out using STATISTICA 13.3 software (Statsoft, Kraków, Poland).

## 3. Results

Table 1 shows the results of the comparison between groups that required (patients on NIRS) and did not require (patients without NIRS) NIRS during hospitalisation.

Patients who required NIRS were significantly older and had more severe changes in the lung parenchyma; their hospitalisation time was also longer. They had higher white blood cell and neutrophil counts and lower lymphocyte counts. The use of these therapies was also associated with higher concentrations of D-dimer, CRP, PCT, and IL-6 and greater activities of LDH and AST in the blood.

Overall, 101 patients required NIRS. The health condition of 47 patients improved (NIRS-effective group), while in 54 patients the use of these methods of oxygen therapy and/or ventilation support proved insufficient (NIRS-ineffective group). In total, 31 patients required intubation and transfer to the ICU, and 23 died of sudden causes or were disqualified from intensification of treatment within the ICU. Only three patients admitted to the ICU were discharged from the hospital. They were all alive at the end of the follow-up period of this study. Additionally, two patients died suddenly—they were not admitted to the ICU, nor did they receive NIRS.

Table 2 presents a comparison between the NIRS-effective and NIRS-ineffective patients. Failure of NIRS was defined as the patient’s death or the need for intubation and transfer to the ICU.

NIRS-ineffective patients were significantly older compared with NIRS-effective patients. They had lower white blood cell and neutrophil counts, as well as higher creatinine concentration and AST activity. There was no significant difference between the groups in the involvement of the lung parenchyma by inflammatory changes. In both groups, the median value of lung parenchyma involvement was found to be high, at 60% in the NIRS-ineffective group and 65% in the NIRS-effective group.

Using univariable logistic regression analysis, we identified factors that significantly increased the risk of the need to use NIRS. The results of the analyses are presented in Table 3.

The factor that increased the risk of the need to use advanced methods of oxygen therapy more than four times was the involvement of the lung parenchyma by inflammatory changes on computed tomography above 50%.

Using multivariable logistic regression analysis, the model with the greatest ability to predict the need for NIRS included inflammatory changes in CT involving ≥50% of the lung parenchyma, increased PCT and WBC, and decreased lymphocyte count.

**Figure 1:**
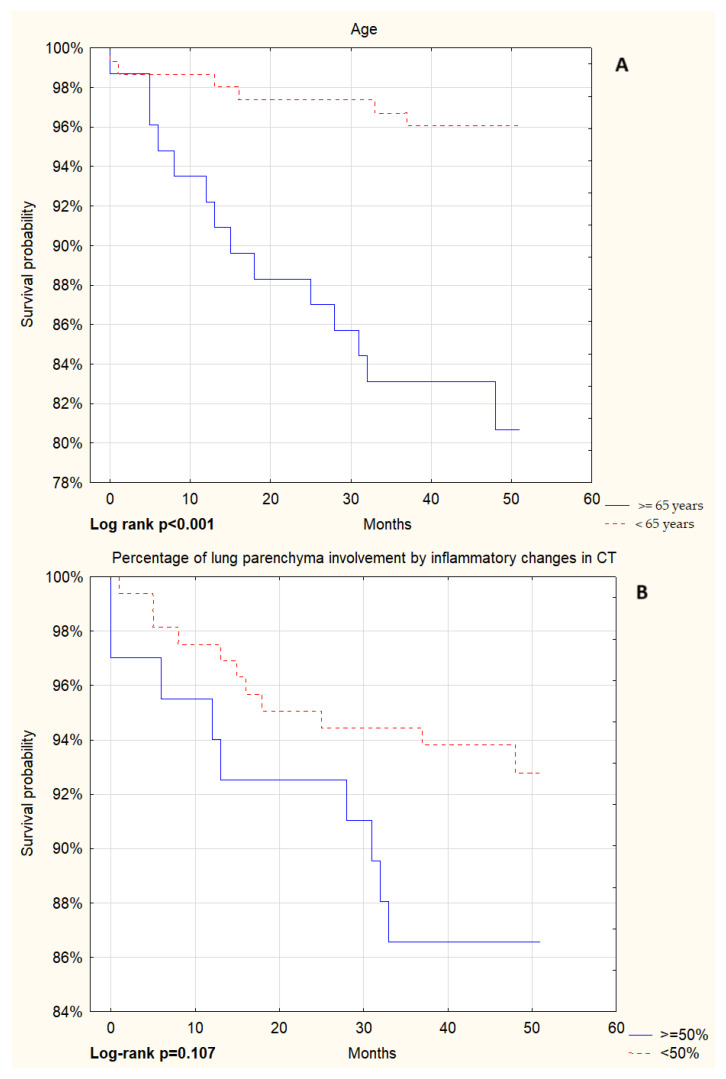

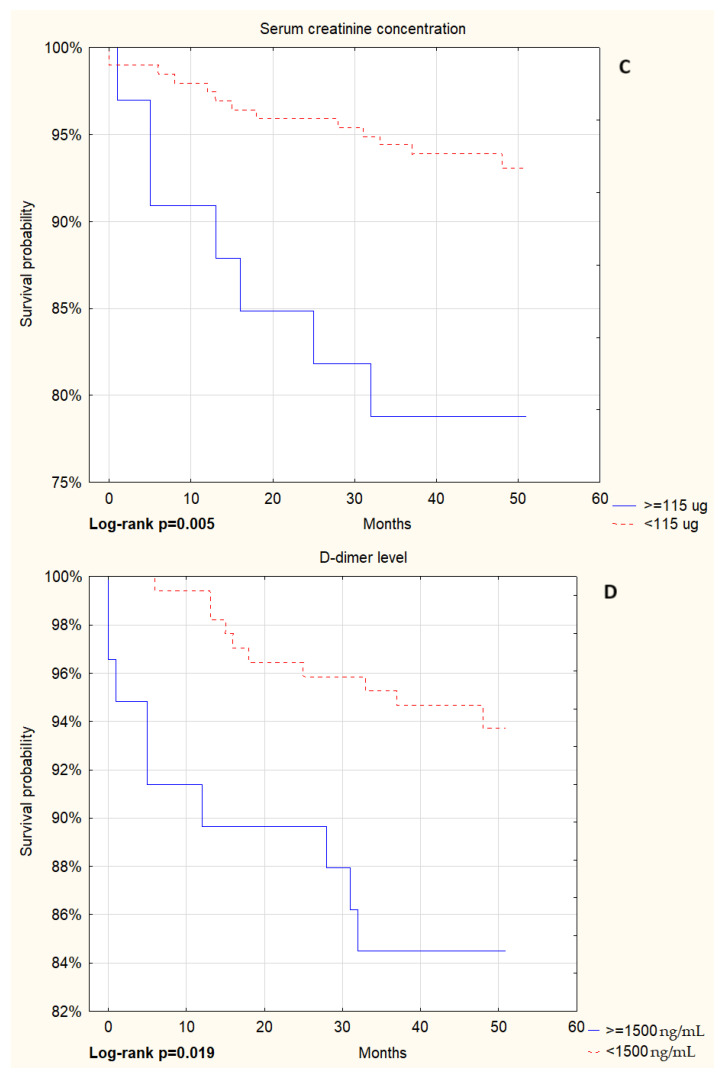

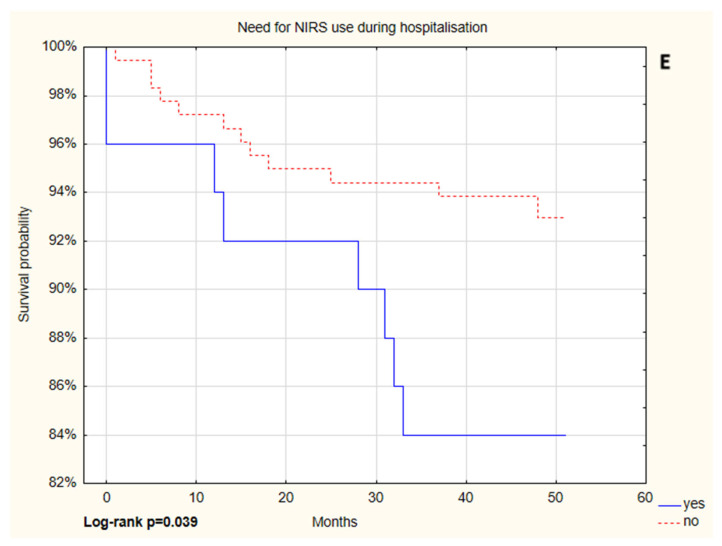
Kaplan–Meier survival curves of all live patients discharged from the hospital after hospitalisation due to COVID-19 for various predictors. The figure shows Kaplan–Meier survival plots by (**A**) age, (**B**) percentage of lung parenchyma involvement by inflammatory changes on CT, (**C**) serum creatinine concentration, (**D**) D-dimer level, and (**E**) need for NIRS use during hospitalisation.

A total of 229 patients were discharged from the hospital, and by the end of the study 20 (8.7%) had died. Multivariate survival analysis using the Kaplan–Meier method was performed after hospitalisation due to COVID-19. Patients who died were significantly older. The degree of lung parenchyma involvement in HRCT did not affect survival. However, the need for NIRS during hospitalisation, associated with a more severe course of the disease, turned out to be a significant predictor. Laboratory parameters whose increased values were associated with a higher risk of death after hospital discharge were serum creatinine concentration and D-dimer level. The essential results of the statistical analyses are presented in Figure 1.

The results of the multivariate analysis of survival (crude and adjusted hazard ratio by Cox regression model) for death after hospital discharge due to COVID-19 are presented in Table 4. The model that showed the highest predictive value included two variables: age and the need for NIRS during hospitalisation.

## 4. Discussion

Currently, mainly due to vaccinations, severe cases of COVID-19 are less common. However, they still constitute a large burden on the healthcare system. It is crucial to identify patients at risk of severe SARS-Co-2 infection at first contact with the healthcare system. This will allow for early identification of patients who will require hospitalisation in specialist departments with appropriate medical equipment and access to the ICU. In our study, we enrolled patients admitted to the Pulmonology Department of the COVID Temporary Hospital. We defined patients at risk of severe SARS-CoV-2 infection as those requiring the use of NIRS. We hope that the results will help clinicians in the early identification of patients at the highest risk of severe disease and in providing even more effective treatment.

The indicator that showed the greatest predictive value for the need to use NIRS was the degree of involvement of the lung parenchyma on CT imaging by inflammatory changes of various morphology. Similar analyses have already been conducted in many studies, but they focused on the degree of severity of inflammatory changes on CT and the risk of intubation or patient death [20,21,22,23,24]. In the studies conducted by Zhao et al. and Li et al., bilateral occurrence of changes in the chest CT image was considered a prognostic factor for a severe course. However, the focus was not on the percentage of affected lung parenchyma [25,26]. Seead et al. showed that the severity of changes on HRCT of the lungs presented a positive correlation with the severity of the patient’s condition and the length of hospital stay. In addition, it was found that the severity of changes on CT correlated positively with lymphopenia, increased serum CRP concentration, D-dimer, and ferritin levels [27]. Direct comparison between the above studies and our research results is difficult, due to the different endpoints of the studies. The conclusion that is common to all analyses emphasises that the severity of changes on chest HRCT showed a positive correlation with the severity of the course of SARS-CoV-2 infection.

Patients who required NIRS were significantly older compared to those who did not require this therapy. Older age was found to be among the predictors of NIRS failure. Previous studies have already shown that older age was an unfavourable prognostic factor, and these results were also confirmed in previous studies of the Polish population [20,24,25,28,29].

Our results showed that age is an important factor leading to NIRS failure, which can be explained by the fact that there is chronic, mild inflammation. This phenomenon has been termed “inflammaging”, is associated with both morbidity and mortality, and increases with age [30]. Other studies have also confirmed the influence of age on the reduced effectiveness of HFNOT and NIRS [31,32].

Patients included in our study who required NIRS and presented a more severe course of disease had higher white blood cell and neutrophil counts and lower lymphocyte counts. Similar to a study by Zhu et al., the white blood cell count was higher in patients with a more severe course of disease [33]. However, similar to our results, the median leukocyte level was within norms for both groups of patients.

Higher white blood cell and neutrophil counts and lower lymphocyte counts were also found in patients who had a more severe course of infection in a study conducted in Wunan, China [34,35] and in a narrative review performed by Palladino [36]. Patients in whom the use of NIRS proved to be significantly insufficient for improvement of health had lower white blood cell and neutrophil counts. This is in contradiction with other studies; however, the statistical significance is not high and may be due to the small size of the groups. Patients for whom the use of NIRS was ineffective had lower leukocyte and neutrophil counts, which may be attributed to their admission at a more advanced stage of the disease or a lower response of specific defence mechanisms.

In a study by Reyes et al., patients treated with HFNOT, non-invasive ventilation (NIV), or IMV within the first 24 h of hospital admission were included. In the first 24 h of hospital admission, patients in high-income countries were more likely to be treated with HFNOT (48.0%), followed by NIV (38.6%) and IMV (13.4%). Higher leukocyte counts and tachypnoea were risk factors for NIRS failure [37]. This study differs in methodology from our research, because only patients who required NIRS within 24 h of hospitalisation were included in that study. However, it is noteworthy that a prediction of NIRS efficacy could be made based on leukocyte counts. In a study conducted by Varpaei et al., patients in whom NIV was ineffective had significantly higher LDH activity compared to patients in whom NIV was sufficient to improve their health. In this study, no differences in CRP levels were found between these groups [38]. In our study, we did not find any differences in CRP and LDH values between the NIRS-effective and NIRS-ineffective groups.

In a study conducted in an Italian population, increased LDH activity, increased ALT activity, increased CRP level, and D-dimer level > 1000 ng/mL were found to be significantly associated with the use of NIRS or invasive ventilation. However, in this study, older age was not found to have a negative effect on the need for ventilatory support [39].

There was no difference in creatinine levels between the groups requiring and not requiring NIRS. However, the group of patients in whom NIRS was ineffective had significantly higher creatinine levels. In a study by Dyrbuś et al., the presence of chronic kidney disease was a predictor of the need for active oxygen therapy, but also of all-cause mortality [40]. Previous studies have also shown that the presence of kidney disease is associated with a more severe course of infection [41].

PCT is a recognised marker of bacterial infection [42]. However, many studies have also found that its elevated level is an indicator of a severe course of SARS-CoV-2 infection [43,44]. In our study, patients requiring NIRS had a higher level of PCT. However, this laboratory parameter did not demonstrate usefulness in identifying patients in whom NIRS would not be sufficient to improve their health.

A study from South Korea showed that the risk of all-cause mortality after COVID-19 is elevated for up to 6 months, then decreases significantly and returns to normal within 1 year [45]. In our study, age 65 years and above was the most significant predictor of death, but it is also a strong predictor of death in the general population. Novelli et al. showed that severe pneumonia in the course of SARS-CoV-2 infection was not a significant predictor of mortality after hospital discharge [18]. Conversely, our study identified the need for NIRS as a significant predictor of post-hospital mortality. Additionally, we observed that elevated serum creatinine levels at the time of hospital admission were associated with reduced survival after discharge. This finding was already present in previous studies, where chronic kidney disease was found to increase the risk of post-hospital mortality [46].

### 4.1. Strengths of the Study

This study presents the characteristics of a Polish population infected with SARS-CoV-2, which required the use of NIRS when COVID-19 was diagnosed. To our knowledge, this is one of the few studies that identifies prognostic factors based on imaging and laboratory test results obtained at hospital admission to predict the need for NIRS during hospitalisation. The results of laboratory parameters and the performance of HRCT of the lungs are available even in hospitals with a lower reference level, so it is a study that is relevant to all hospitals in the country. Due to the fact that NIRS is still not widely available in all hospitals, and that its use requires specialist knowledge and experience, the identification of patients in whom it will be necessary to use is already crucial at the early stage of hospitalisation. We were able to conduct a long-term analysis of patient mortality after hospital discharge and identify its predictors.

### 4.2. Limitations of the Study

Limitations of the present study are the retrospective nature, the single-centre nature, and the small sample size, which limit the generalisability of the results. The small number of patients results from the adopted exclusion criteria. Only patients with full data on the oxygen therapy used and the results of laboratory and imaging tests, who had been staying at the Pulmonology Department of the Temporary Hospital from the beginning of their hospitalisation, were qualified for the study. We did not have full access to the results of patients’ blood gas tests. In our study, we did not analyse the chronic diseases of the patients. This resulted from our observations that a significant proportion of patients who were admitted to hospital had not previously been diagnosed with their diseases. Therefore, at the early stage of admission to the hospital, it was not known that they had them, and these diseases were often diagnosed in the later stages of hospitalisation. Since the analysis focused on identifying patients at risk of severe disease upon hospital admission, we also did not analyse the treatment used so far, the SARS-CoV-2 variants, or the vaccination status.

A certain limitation of our study may be the lack of identification of specific changes involving the lung parenchyma on CT images. However, due to the assumption of this study, the main goal of which was to quickly identify consecutive patients at risk of severe SARS-CoV-2 infection, we considered that determining the percentage of inflammatory changes in the lung parenchyma alone would be a sufficient, simple, and quick prognostic factor.

## 5. Conclusions

In conclusion, more than 50% involvement of the lung parenchyma by post-infectious lesions on chest CT, leucocytosis with lymphopenia, and PCT elevation above the normal range were significantly associated with the need for NIRS. Laboratory tests and chest computed tomography performed on hospital admission may be useful to rapidly identify patients at higher risk for severe disease and to treat them appropriately. Large-scale randomised controlled trials are needed to confirm and extend our findings.

Patients aged 65 years and older with higher levels of D-dimer and serum creatinine concentration were characterised by a higher risk of death after hospital discharge. More severe disease associated with the occurrence of respiratory failure and the need for NIRS also increased the risk of death after hospitalisation.

## Figures and Tables

**Table 1:** Comparison between patients requiring NIRS during hospitalisation and those not requiring this therapy.

Parameter	Patients Without NIRS (N = 181)	Patients on NIRS (N = 101)	*p* Value
Average length of hospitalisation in the department [days]	7 (5–10)	13 (8–18)	<0.001
Age [years]	58 (46–67)	66 (60–72)	<0.001
Lung parenchyma percentage involvement in CT [%]	25 (15–40)	60 (50–70)	<0.001
Duration of oxygen therapy [percentage of hospitalisation time]	63 (0–81)	100 (93–100)	<0.001
WBC [10^9^/L]	6.1 (4.5–7.8)	8.1 (6.1–10.3)	<0.001
NEUT [10^9^/L]	4.7 (3.0–6.2)	7.0 (5.01–9.2)	<0.001
LYMPH [10^9^/L]	0.8 (0.6–1.2)	0.6 (0.5–0.8)	<0.001
ALT [U/L]	39.0 (25.0–60.0)	46.0 (29.0–66.0)	0.25
AST [U/L]	43.0 (30.0–63.0)	58.0 (44.0–97.0)	<0.001
Creatinine [umol/L]	83.0 (70.0–100.0)	89.0 (70.0–115.0)	0.06
D-dimer [ng/mL]	706.0 (484.0–1221.5)	1417.0 (766.0–5831.0)	<0.001
LDH [U/L]	345.0 (266.0–434.0)	527.0 (407.0–647.0)	<0.001
CRP [mg/L]	67.0 (31.0–122.0)	136.0 (80.0–200.0)	<0.001
PCT [ng/mL]	0.07 (0.04–0.14)	0.19 (0.11–0.38)	<0.001
IL-6 [pg/mL]	25.7 (7.5–47.3)	44.1 (21.2–104.4)	<0.001

Data are presented as median (interquartile range). NIRS—non-invasive respiratory support; CT—computed tomography; WBC—white blood cells; NEUT—neutrophils; LYMPH—lymphocytes; ALT—alanine aminotransferase; AST—aspartate aminotransferase; LDH—lactate dehydrogenase; CRP—C-reactive protein; PCT—procalcitonin; IL-6—interleukin 6.

**Table 2:** Comparison between the NIRS-effective and NIRS-ineffective groups.

Parameter	NIRS-Effective Group (N = 47)	NIRS-Ineffective Group (N = 54)	*p* Value
Hospitalisation time in the department [days]	17 (12–24)	9 (4–13)	<0.001
Age [years]	64 (55–68)	69 (63–79)	<0.001
Lung parenchyma involvement on CT [%]	65 (55–70)	60 (45–70)	0.09
Duration of oxygen therapy [percentage of hospitalisation time]	91.7 (83.3–100.0)	100.0	<0.001
WBC [10^9^/L]	9.0 (6.5–11.3)	7.5 (5.4–9.2)	0.042
NEUT [10^9^/L]	7.8 (5.6–10.1)	6.2 (3.9–8.2)	0.034
LYMPH [10^9^/L	0.6 (0.5–0.9)	0.5 (0.4–0.8)	0.62
ALT [U/L]	47.0 (29.0–71.0)	46.0 (25–62)	0.71
AST [U/L]	55.0 (36.0–82.0)	67.5 (49.0–109.0)	0.041
Creatinine [umol/L]	83.0 (62.0–108.0)	92.0 (80.0–129.0)	0.012
D-dimer [ng/mL]	1426.0 (668.0–5357.0)	1417.0 (886.0–6768.0)	0.74
LDH [U/L]	478.0 (390.0–644.0)	559.5 (436.0–665.0)	0.16
CRP [mg/L]	155.0 (79.0–272.0)	125.0 (80.0–191.0)	0.35
PCT [ng/mL]	0.21 (0.1–0.36)	0.17 (0.11–0.28)	0.93
IL-6 [pg/mL]	43.4 (19.9–104.4)	44.1 (21.2–88.7)	0.71

Data are presented as median (interquartile range). NIRS—non-invasive respiratory support; IMV—invasive mechanical ventilation; CT—computed tomography; WBC—white blood cells; NEUT—neutrophils; LYMPH—lymphocytes; ALT—alanine aminotransferase; AST—aspartate aminotransferase; LDH—lactate dehydrogenase; CRP—C-reactive protein; PCT—procalcitonin; IL-6—interleukin 6.

**Table 3:** Univariate and multivariable logistic regression model for the factors associated with the need to use NIRS.

	Univariable		Multivariable	
Variable	Odds Ratio (95% of CI)	*p* Value	Odds Ratio (95% of CI)	*p* Value
Inflammatory changes in CT involving ≥50% of the lung parenchyma	4.52 (3.30–6.18)	<0.001	4.38 (3.06–6.28)	<0.001
Gender (male)	0.88 (0.53–1.48)	0.63		
Age ≥ 65 years	1.60 (1.24–2.05)	<0.001		
WBC ≥ 10 × 10^9^/L	1.85 (1.35–2.55)	<0.001	1.68 (1.07–2.64)	0.023
NEUT ≥ 7 × 10^9^/L	2.18 (1.66–2.87)	<0.001		
LYMPH ≤ 0.5 × 10^9^/L	1.91 (1.41–2.58)	<0.001	1.50 (1.00–2.24)	0.051
AST ≥ 45 U/L	1.74 (1.34–2.27)	<0.001		
Creatinine ≥ 115 umol/L	1.44 (1.06–1.95)	0.024		
D-dimer ≥ 1500 ng/mL	2.04 (1.56–2.68)	<0.001		
LDH ≥ 350 U/L	2.63 (1.90–3.65)	<0.001		
CRP ≥ 80 mg/L	2.05 (1.57–2.69)	<0.001		
PCT ≥ 0.1 ng/mL	2.62 (1.96–3.50)	<0.001	2.27 (1.56–3.31)	<0.001
IL-6 ≥ 40 pg/mL	1.64 (1.27–2.11)	<0.001		

NIRS—non-invasive respiratory support; CT—computed tomography; NS—not significant; WBC—white blood cells; NEUT—neutrophils; LYMPH—lymphocytes; AST—aspartate aminotransferase; LDH—lactate dehydrogenase; CRP—C-reactive protein; PCT—procalcitonin; IL-6—interleukin 6.

**Table 4:** Multivariate survival (crude and adjusted hazard ratio by Cox regression model) for death after hospitalisation due to COVID-19.

Variables		*p* Value	HR * (95% of CI)	*p* Value	HR ** (95% of CI)
Age		<0.001	1.08 (1.04–1.12)	0.001	1.09 (1.04–1.13)
NIRS	no		1		1
	yes	0.043	2.51 (1.03–6.15)	0.013	2.53 (1.03–6.26)

NIRS—non-invasive respiratory support; * crude HR; ** adjusted HR.

## Data Availability

The data presented in this study are available on request from the corresponding author.

## Abbreviations

The following abbreviations are used in this manuscript:

SARS-CoV-2	Severe acute respiratory syndrome coronavirus 2
COVID-19	Coronavirus disease 2019
NETs	Neutrophil extracellular traps
HFNOT	High-flow nasal oxygen therapy
CPAP	Continuous positive airway pressure
BPAP	Bilevel positive airway pressure
NIRS	Non-invasive respiratory support
IMV	Invasive mechanical ventilation
PCR	Real time polymerase chain reaction test
CT	Computed tomography
AST	Aspartate aminotransferase
ALT	Alanine aminotransferase
LDH	Lactate dehydrogenase
CRP	C-reactive protein
PCT	Procalcitonin
IL-6	Interleukin-6
CBC	Complete blood count
HRCT	High resolution computed tomography
ICU	Intensive Care Unit
NS	Not significant
WBC	White blood cells
NEUT	Neutrophils
LYMPH	Lymphocytes
NIV	Non-invasive ventilation
